# Molecular mechanisms of vitamin D plus Bisphenol A effects on adipogenesis in human adipose-derived mesenchymal stem cells

**DOI:** 10.1186/s13098-021-00661-4

**Published:** 2021-04-09

**Authors:** Amin Salehpour, Farzad Shidfar, Mehdi Hedayati, Ali Asghar Farshad, Asal Neshatbini Tehrani, Saeed Mohammadi

**Affiliations:** 1 Occupational Health Research Center, School of Public Health, Iran University of Medical Sciences, Shahid Hemmat Highway, 1449614535 Tehran, Iran; 2 Cellular and Molecular Endocrine Research Center, Research Institute for Endocrine Sciences, Shahid Beheshti University of Medical Sciences, Tehran, Iran; 3 Department of Nutrition, School of Paramedical, Ahvaz Jundishapur University of Medical Sciences, Ahvaz, Iran; 4 Department of Biostatistics, School of Public Health, Iran University of Medical Sciences, Tehran, Iran

**Keywords:** 1,25-Dihydroxyvitamin D3, Bisphenol A, Adipogenesis, Human adipose-derived mesenchymal stem cells, Gene expression

## Abstract

**Background:**

Obesity is considered a major health concern and mounting evidence suggests that the exposure to environmental endocrine disruptors, including Bisphenol-A (BPA), may enhance the risk to develop the disease. Moreover, growing documents propose that the vitamin D may contribute to adipogenic signaling and lipid accumulation during adipocyte differentiation. We focused on the molecular mechanism of vitamin D and BPA in human adipose-derived mesenchymal stem cells (hADMSCs) which vitamin D and BPA may influence adipose tissue development and function.

**Methods:**

Human adipose-derived mesenchymal stem cells were cultured for 14 days in lipogenic differentiation media containing continuous concentrations of vitamin D plus BPA (0.1 nM or 10 nM). The expression of adipogenic markers including the peroxisome proliferator-activated receptor γ (PPARγ), CCAAT-enhancer-binding protein α (C/EBP α) CCAAT-enhancer-binding protein β (C/EBP β), fatty acid synthase (FASN), lipoprotein lipase (LPL), sterol regulatory element-binding protein-1c (SREBP1c), insulin-induced gene-2 (INSIG2), vitamin D receptor (VDR), estrogen receptor-beta (ER-β), fatty acid-binding protein-4 (FABP4), and glucose transporter-4 (GLUT4) was measured using Quantitative polymerase chain reaction (qPCR) and enzyme-linked immunosorbent assay (ELISA). Lipid accumulation was visualized with staining with Oil Red O.

**Results:**

In the morphological assessment of mesenchymal stem cells treated with a concentration of 10 nM vitamin D plus BPA, more lipid accumulations were observed in comparison with the group with 0.1 nM concentration. Treatment of hADMSCs with vitamin D plus BPA (0.1 nM) significantly inhibited the induction of PPARγ, C/EBP β, C/EBP α, and FASN related to adipocyte differentiation and development. However, the exposure of cells to the concentration of 10 nM vitamin D plus BPA induced the expression of these genes associated to the adipogenesis. The remarkable increase in the level of SREBP1c was associated to the suppression of INSIG2 in treated preadipocytes with 10 nM vitamin D plus BPA. Our findings showed that the expression of VDR, ERβ, GLUT4, and FABP4 were upregulated through differentiation with the highest concentrations in 0.1 nM vitamin D plus BPA group for VDR, ERβ, and GLUT4.

**Conclusions:**

Vitamin D plus BPA at concentration of 10 nM boosted the adipogenesis during the critical stages of adipocytes development, whereas it seems to inhibit this process at concentration of 0.1 nM.

## Background

Obesity is a chronic disease in which the excess adipose tissue has accumulated in the body. Fat accumulation is considerably related to cardiovascular disease, diabetes type 2, hypertension, sleep apnea, musculoskeletal disorders, psychological conditions, and a few cancers. Moreover, obesity is contributed to higher mortality and morbidity rates as well as reduced life expectancy [1, 2].

The regulation of adipose tissue expansion is a remarkable process characterized by the increase in the volume of adipocytes known as hypertrophy and the number of adipocytes known as hyperplasia called adipogenesis. These are necessary for lipid storage and development to form the mature adipocytes [3–5].

The expression of nuclear and membrane vitamin D receptors (VDR) in adipocytes proposes that adipose tissue is responsive to vitamin D. A range of mechanisms by which vitamin D possibly affects adipogenesis and energy balance were suggested [6, 7].

The evidence supports stimulating and suppressing actions of vitamin D on adipogenesis. In mouse models, vitamin D blunted adipogenesis through acting on various targets suppressing the expression of CCAAT-enhancer-binding protein α (C/EBPα) and peroxisome proliferator-activated receptor γ (PPARγ), downregulating the mRNA expression of CCAAT-enhancer-binding protein β (C/EBP β) and its nuclear protein levels in 3T3-L1 preadipocytes [8, 9]. In 3T3-L1 preadipocytes, vitamin D inhibits the adipogenesis by suppressing C/EBPα and PPARγ expression and down-regulating transcription of C/EBPβ and its nuclear protein. Vitamin D also activates Eight-Twenty-One factor (ETO), C/EBPβ co-receptor, which plays more inhibitory role on the C/EBPβ function. WNT/β-Catenin keeps preadipocyte cells in the undifferentiated phase which prevents adipogenesis. Vitamin D exerts the anti-adipogenic effects on 3T3-L1 cells by maintaining the expression levels of nuclear WNT10B and β-Catenin and suppressing PPARγ [9].

In human tissue, vitamin D stimulates the differentiation of subcutaneous preadipocytes by augmenting the expression of fatty acid-binding protein-4 (FABP4), and lipoprotein lipase (LPL) [10, 11]. Vitamin D enhances lipid accumulation and increases expression of adipogenic genes, including fatty acid synthase (FASN), FABP4*,* and PPARγ to differentiate mesenchymal cells to adipocytes [12–14].

The rising prevalence of obesity was preceded by an exponential growth to produce synthetic chemicals which resulted in proposing a new hypothesis called environmental obesogen [15]. Bisphenol A (BPA) is a lipophilic compound characterized as an endocrine disrupter with estrogen mimetic activity. BPA is so widespread in the daily human environment which found in products including polycarbonate plastics, the lining of canned food, beverage, and more. Therefore, it can transfer into human bodies. BPA is detected in human plasma and urine as well as in the human milk [15–19]. The experimental data report that BPA interferes with main weight controlling hormones such as thyroid hormones, estrogen, testosterone, glucocorticoids, and leptin. BPA acts as a potential disrupter to develop obesity by provoking lipid accumulation and differentiating preadipocytes from the mature adipocytes by expressing adipogenic genes such as C/EBP α, FABP4, PPARγ, and LPL [17, 20–22]. BPA in micromole concentrations induces the differentiation of adipocytes, while in nanomol concentrations, it reduces insulin sensitivity in adipose tissue. Decreased Glucose transporter-1 (GLUT-1) expression and insulin receptor phosphorylation impair glucose metabolism and increase the risk of diabetes type 2.

BPA interacts with nuclear estrogen receptors, glucocorticoids, PPARγ, retinoid X receptor (RXR), and thyroid at nuclear micromol concentrations which is all these receptors are involved in adipogenesis [17]. BPA induced adipocyte differentiation and lipid accumulation through activating these receptors [17]. We tested the effects of vitamin D plus BPA on the differentiation of human adipose derived mesenchymal stem cells (hADMSCs) to adipocytes.

## Methods

### Human adipose-derived stem cells

hADMSCs obtained from the human cell bank of Iranian Biological Resource Center laboratory (Tehran, Iran). Written informed consent for using and collecting the cells and tissues was obtained from all donors. Human ASC highly used in all experiments were from subcutaneous abdominal adipose tissue of female donors with an average age of 37 and body mass index (BMI) average of 26.2 kg/m^2^. Dulbecco’s modified Eagle medium (DMEM) supplemented with 10% FBS, and 2% glutamine, 100 IU/ml penicillin, and 100 IU/ml streptomycin were used as growth media which incubated at 37 °C with 5% humidified CO_2_. The cells were passed when they reached 80–90% confluence with media changed in the 2–3-day interval. Once 80–90% confluence was reached (4 passage), all wells were divided into three experimental groups with at least three parallel wells in each group: (i) 0.1 nM of vitamin D plus 0.1 nM of BPA with induction; (ii) 10 nM of vitamin D in the active metabolic form, 1,25-dihydroxyvitamin D3, plus 10 nM BPA with induction; (iii) control with induction and without vitamin D or BPA. To induce the differentiation into mature adipocytes, cells were suspended and cultured in an adipocyte differentiation medium (Gibco, UK) involving the concentrations of 0.5 mM 3-isobutyl-3-methylxanthine (IBMX), 1 mM dexamethasone, 5 mg/ml human insulin for 14 days. After a week, the media were replaced with an adipocyte maintenance medium (Gibco, UK) and cultured for 7 days. Treatment compounds were added at the same concentrations when media was changed. All these mix changes were done in parallel in control cells without vitamin D. Cells were collected and harvested to assess messenger ribonucleic acid (mRNA) adipogenic genes at hours 1, 3, and days 1, 3, 7 and 14 during the differentiation. All chemical materials were purchased from Sigma-Aldrich.

### Quantitative real-time reverse transcription-PCR (qRT-PCR)

The mRNA level of adipogenesis-related genes, PPARγ, C/EBPα, C/EBPβ, LPL, FASN, sterol regulatory element-binding protein-1c (SREBP-1c), Insulin-induced gene 2 (INSIG2) were quantified by qRT-PCR assay. After a 2-week adipogenic-induction, total RNA was extracted from differentiating cells treated using TRIzol reagent (Sigma-Aldrich) due to the manufacturer’s instruction, and then RNA was reverse transcribed into cDNA using Superscript II reverse transcriptase kit (Invitrogen) following manufacturer’s protocol. Quantitative PCR analysis was performed using StepOnePlus PCR System (Applied Biosystems) and SYBR Premix Ex Taq II, Tli RNaseH Plus (Takara, Japan). Fold changes in gene expression level were calculated by 2^−∆∆Ct^ method, and the results were normalized to the expression of an internal control, Glyceraldehyde-3-Phosphate Dehydrogenase (GAPDH). PCR primer sequences were listed as followed, with primer specificity confirmed on NCBI Primer-BLAST website:

PPARγ: Forward 5′-CAGAAATGCCTTGCAGTGGG-3′, Reverse 5′-AACAGCTTCTCCTTCTCGGC-3′; C/EBPα: Forward 5′-TATAGGCTGGGCTTCCCCTT-3′, Reverse 5′-AGCTTTCTGGTGTGACTCGG-3′; C/EBPβ: Forward 5′-TTTGTCCAAACCAACCGCAC-3′, Reverse 5′-GCATCAACTTCGAAACCGGC-3′; SREBP1c: Forward 5′-TCTCAGTCCCCTGGTCTCTG-3′, Reverse 5′-ATAGGCAGCTTCTCCGCATC-3′; insig-2: Forward 5′-AGTGGTCCAGTGTAATGCGG-3′, Reverse 5′-TGGATAGTGCAGCCAGTGTG-3′; LPL: Forward 5′-GCTCAGGAGCATTACCCAGTGTC, Reverse 5′-GCTCCAAGGCTGTATCCCAAGA-3′; FASN: Forward 5′-ATTCTGCCATAAGCCCTGTC-3′, Reverse 5′-CTGTGTACTCCTTCCCTTCTTG-3; and GAPDH: Forward 5′-CATGAGAAGTATGACAACAGCCT-3′, Reverse 5′-AGTCCTTCCACGATACCAAAGT-3′.

### Oil Red O staining

To evaluate the cellular neutral lipid droplet accumulation, mature adipocytes were washed three times with iced phosphate-buffered saline (PBS) which fixed with 4% paraformaldehyde for 30 min. After fixation, the cells were washed three times and stained with Oil Red O solution (ORO) for 15 min at room temperature. Cells were washed three times again with PBS to remove unbound staining. ORO-stained adipocytes were observed under a microscope (Olympus, Tokyo, Japan), and digital images were captured at 100× magnification.

### Protein assay

To determine protein concentration, the plated cells were lysed in a buffer involving 50 mM Tris, 150 mM sodium chloride (NaCl), 1% IGEPAL, 5 mM EDTA (Sigma-Aldrich), protease inhibitor cocktail (Roche Diagnostics, Laval, QC, Canada), and centrifuged for lysate collection. Using specific enzyme-linked immunosorbent assay (ELISA) kits (Human FABP4 ELISA kit, Human GLUT4 ELISA kit, Human ERβ ELISA kit, purchased from ZellBio GmbH, Ulm, Germany), and a microplate reader (Epoch Model, BioTek, Vermont, USA). The intra assays precision by the coefficient of variation percent (CV %) related to each ELISA kit were 5.5, 5.8, 6.1, and 5.9 percent, respectively.

### Statistics

Data are expressed as means ± standard deviation (SD). The mRNA expressions were determined by analysis of variance (ANOVA) with repeated measures and 2-tailed Student t-tests (SPSS 25 for Windows, standard version; SPSS Inc., Chicago, IL, USA).Using GraphPad (GraphPad Software) and the protein expressions were determined by Kruskal–Wallis one-way analysis of variance and Dunn’s multiple comparison test. Means were considered statistically different when p values were less than 0.05.

## Results

**Fig. 1:**
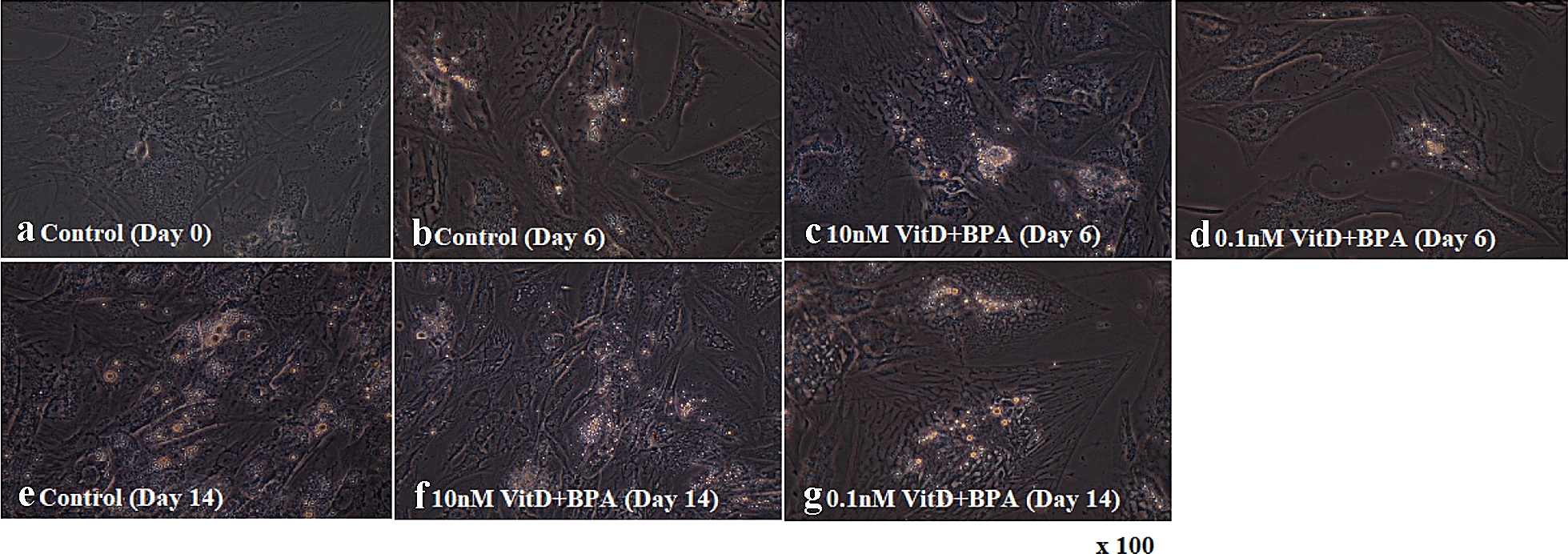
Oil Red O staining of human adipose-derived mesenchymal stem cells. Phase contrast image of adipocytes were taken by microscope (Olympus, Tokyo, Japan) and digital images were captured at ×100 magnification. Following 14 days of treatment with vitamin D plus BPA showed a significant increase in relative lipid vacuole staining compared with control group

The morphology of human adipose-derived mesenchymal stem cells and lipid accumulation were indicated during the differentiation (Fig. 1). More lipid accumulations were observed in control and mesenchymal stem cells treated by 10 nM concentrations of vitamin D plus BPA compared to 0.1 nM vitamin D plus BPA.

**Fig. 2:**
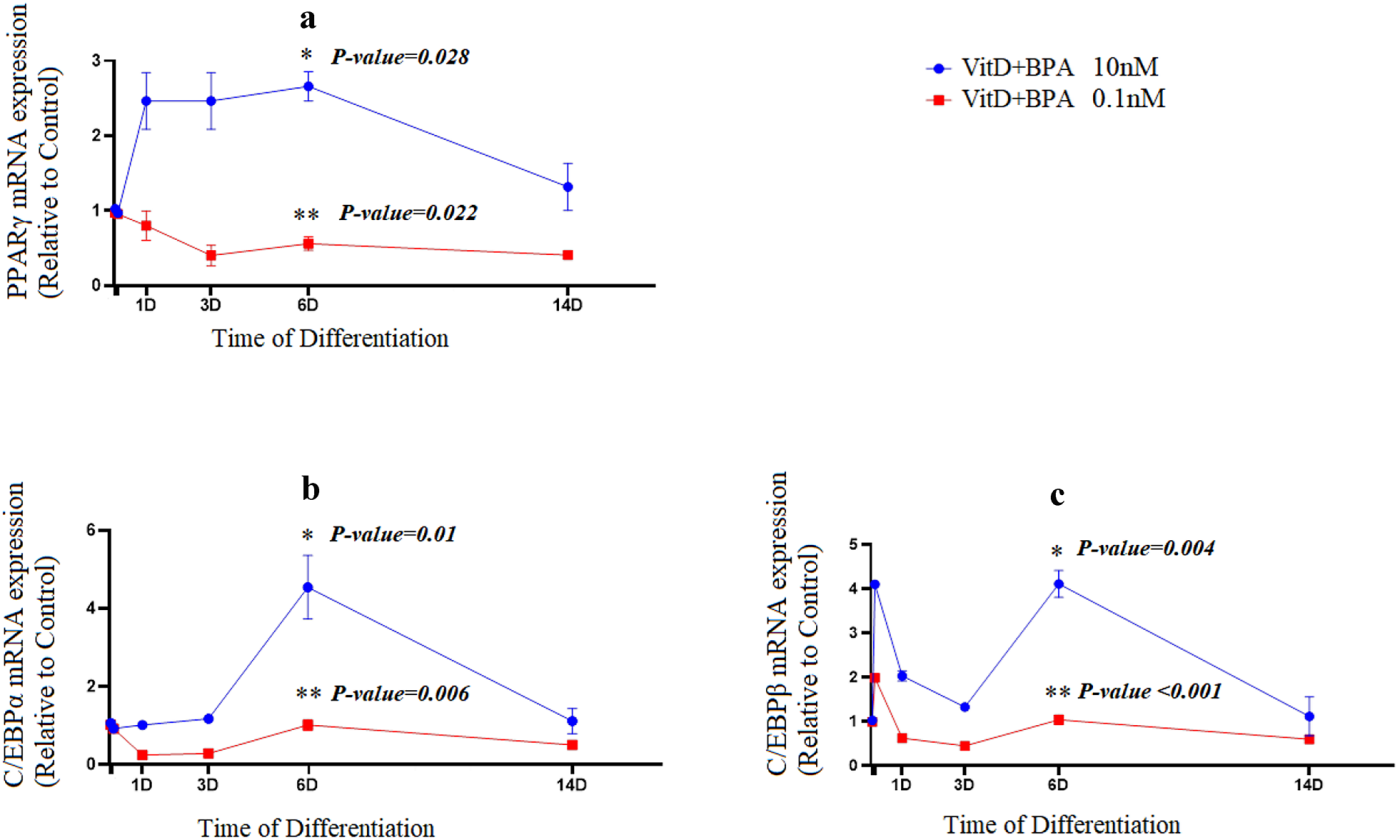
Vitamin D plus BPA modulates the mRNA expression of adipogenic marker genes during adipogenesis dose-dependently. mRNA expression of PPARγ (**a**), C/EBPα (**b**) and C/EBPβ (**c**) in vitamin D plus BPA (10 nM or 0.1 nM) groups during adipogenic differentiation. The relative qPCR values were corrected to GAPDH expression levels and normalized with respect to controls on each time. The mRNA levels are expressed as the fold increase relative to the control

To determine vitamin D plus BPA induced effects on adipogenesis, the mRNA expression of key adipogenic markers and transcription factors were evaluated using qPCR. Vitamin D plus BPA (10 nM) enhanced expression of PPARγ mRNA (P = 0.028) in the early phase of this experiment. In contrast, mRNA expression of PPARγ was suppressed (P = 0.022) through differentiation in response to 0.1 nM vitamin D plus BPA (Fig. 2a).

To evaluate whether vitamin D plus BPA affect early or late adipogenesis events, we next assessed the time course effects on mRNA levels of key transcription factors during differentiation. C/EBPα expression was significantly increased at 6 days post-treatment in response to 10 nM vitamin D plus BPA (P = 0.01), the treatment of cells with 0.1 nM vitamin D plus BPA resulted in a statistically significant reduction (P = 0.006) in the mRNA levels of C/EBPα during differentiation (Fig. 2b). While, the mRNA expression of C/EBPβ, an early adipogenic transcription factor, was inhibited (P < 0.001) with vitamin D plus BPA (0.1 nM), a statistically significant enhanced expression was observed on day 6 (P = 0.004) in the treated cells with vitamin D plus BPA (10 nM) (Fig. 2c).

**Fig. 3:**
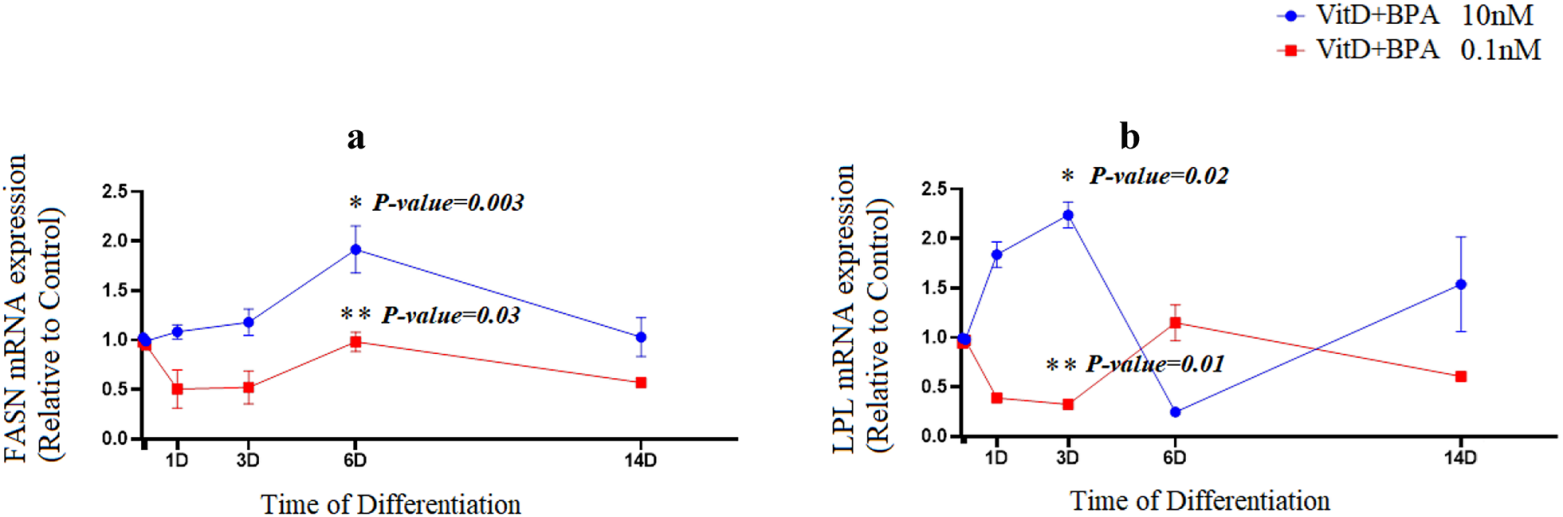
Vitamin D plus BPA (10^−8^ M) augmented mRNA expression of FASN and LPL in adipocytes. mRNA expression of FASN (**a**) and LPL (**b**) in vitamin D plus BPA groups during adipogenic differentiation. The relative qPCR values were corrected to GAPDH expression levels and normalized with respect to controls on each time. The mRNA levels are expressed as the fold increase relative to the control

The levels of FASN mRNA, a marker of de novo lipogenesis, were significantly augmented on day 6 from the beginning of the differentiation process of adipogenesis in cells treated by 10 nM vitamin D plus BPA (P = 0.003); however a reduction was markedly observed (P = 0.03) in 0.1 nM vitamin D plus BPA group (Fig. 3a). Vitamin D plus BPA (0.1 nM) treatment resulted in a suppression of LPL mRNA expression (a late marker of adipogenesis) at day 3 (P = 0.01). While the expression of LPL mRNA had the fluctuation with an increase (P = 0.02) during the early period of differentiation (day 3) (Fig. 3b). Following vitamin D plus BPA (10 nM) exposure, mRNA expression of SREBP1c was significantly increased (P = 0.005) during adipogenesis. Moreover, SREBP1c mRNA levels were significantly prohibited upon vitamin D plus BPA (0.1 nM) exposure (P = 0.034).

**Fig. 4:**
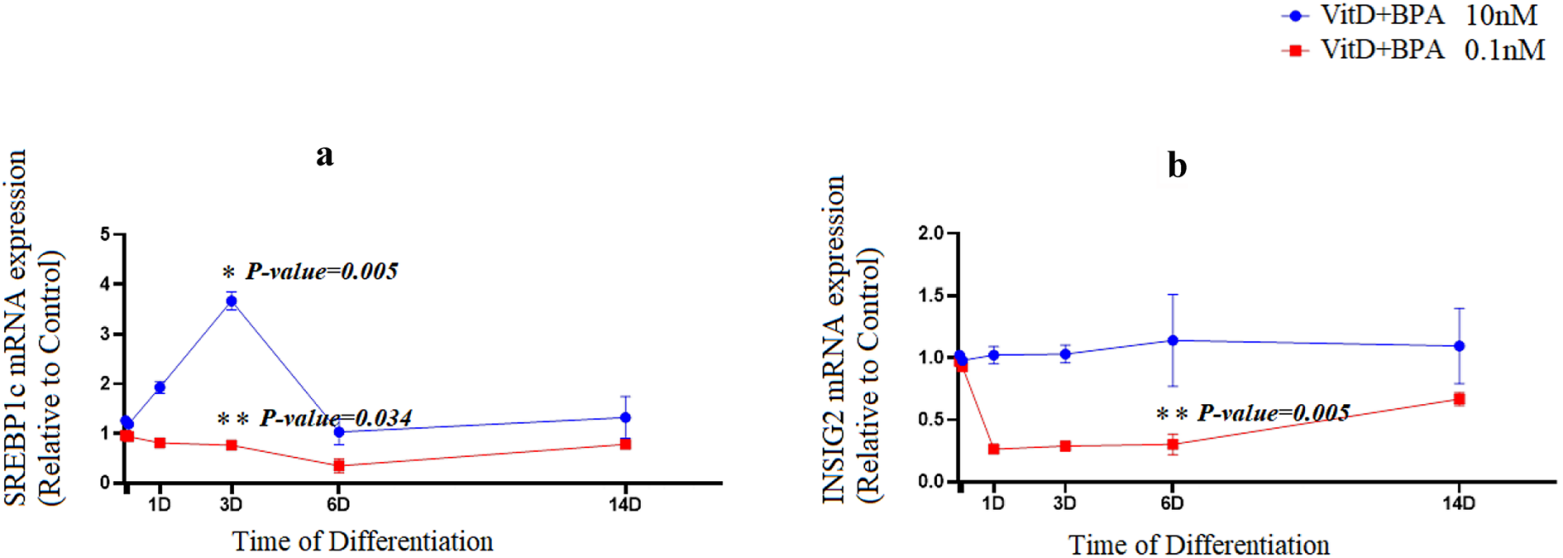
Vitamin D plus BPA differentially regulates the mRNA expression of SREBP1c and INSIG2 during adipogenesis. mRNA expression of SREBP1c (**a**) and INSIG2 (**b**) in vitamin D groups during adipogenic differentiation. The relative qPCR values were corrected to GAPDH expression levels and normalized with respect to controls on each time. The mRNA levels are expressed as the fold increase relative to the control

The treatment with 0.1 nM of vitamin D plus BPA significantly repressed mRNA levels of INSIG2 (P = 0.005), an intermediate regulator between PPARγ and SREBP1c (Fig. 4b). However, the expression levels of INSIG2 were not significantly affected by 10 nM of vitamin D plus BPA (Fig. 4b).

Afterwards, vitamin D effect plus BPA treatment on the protein levels of FABP4, other late markers of adipogenesis, was assessed. The findings showed that protein levels of FABP4 were statistically significant increased in control and vitamin D plus BPA (10 nM) groups, not in the concentration of 0.1 nM vitamin D plus BPA (Table 1). Furthermore, the protein level of GLUT4 was gradually augmented with higher concentration upon 0.1 nM vitamin D plus BPA exposure (Table 1).

**Table 1:** Comparison of protein expression in vitamin D plus BPA groups vs. control

Proteins	Time	Group	Meana	Standard deviation	Result
FABP4	Day 6	Control	0.27	0.03	X = 6.5
		10 nM VitD + BPA	0.19	0.04	df = 2
		0.1 nM VitD + BPA	0.04	0.008	P-value = 0.038*
	Day 14	Control	0.32	0.02	X = 9. 04
		10 nM VitD + BPA	0.32	0.05	df = 2
		0.1 nM VitD + BPA	0.04	0.008	P-value = 0.01*
GLUT4	Day 6	Control	0.17	0.03	X = 9.75
		10 nM VitD + BPA	0.12	0.02	df = 2
		0.1 nM VitD + BPA	0.26	0.02	P-value = 0.007*
	Day 14	Control	0.20	0.01	X = 7. 80
		10 nM VitD + BPA	0.20	0.03	df = 2
		0.1 nM VitD + BPA	0.29	0.04	P-value = 0.02*
VDR	Day 6	Control	0.69	0.45	X = 5.02
		10 nM VitD + BPA	0.49	0.05	df = 2
		0.1 nM VitD + BPA	1.06	0.08	P-value = 0.08
	Day 14	Control	0.80	0.05	X = 8. 05
		10 nM VitD + BPA	0.82	0.05	df = 2
		0.1 nM VitD + BPA	1.12	0.17	P-value = 0.017*
ERβ	Day 6	Control	0.69	0.45	X = 5.02
		10 nM VitD + BPA	0.79	0.18	df = 2
		0.1 nM VitD + BPA	1.70	0.32	P-value = 0.08
	Day 14	Control	0.80	0.05	X = 8. 05
		10 nM VitD + BPA	1.32	0.71	df = 2
		0.1 nM VitD + BPA	2.00	0.56	P-value = 0.017*

Expression levels of adipogenic markers [GLUT4 protein and FABP4 protein] were increased in vitamin D plus BPA (10 nM) group, compared to control and concentration of 0.1 nM vitamin D plus BPA. In addition, protein expression of VDR and ERβ in both concentrations of vitamin D plus BPA vs. control was significantly increased

*FABP4* fatty acid binding proteins-4, *GLUT4* glucose transporter-4, *VDR* vitamin D receptor, *ERβ* estrogen receptor beta

*Kruskal–Wallis test was used and Mean values were significantly different between the groups (P < 0.05)

^a^ng/mg total protein

To address whether VDR or ERβ are involved in the action of vitamin D and BPA during human preadipocyte differentiation, protein levels of VDR and ERβ were measured. Vitamin D plus BPA dose-dependently enhanced VDR and ERβ expression levels compared to the control group (Table 1).

## Discussion

For the first time and to the best of our knowledge, we tested the collective effects of vitamin D and BPA on adipogenesis in hADMSCs. Our results provide original insights into vitamin D and BPA actions on adipogenic differentiation of human adipose-derived stem cells. Vitamin D and BPA was added to the culture before differentiation started to investigate the impact of early exposure on undifferentiated cells. We observed that exposure to vitamin D plus BPA modulates adipocyte differentiation by altering the expression of master adipogenic markers. The expression of genes associated to adipogenic differentiation, including PPARγ, C/EBPα, C/EBPβ, FASN, LPL, and SREBP1c, was significantly enhanced through treatment with 10 nM vitamin D plus BPA. However, the expression of these genes was inhibited in cells treated to 0.1 nM vitamin D plus BPA. Besides, the expression level of proteins, including VDR, ERβ, and FABP4, was augmented. Since many researches have confirmed the effect of BPA to induce the adipogenic differentiation [17, 19, 20], this difference in gene expression is related to the effect of vitamin D [23, 24]. Moreover, we observed inhibited lipid accumulation in mature adipocytes treated with 0.1 nM vitamin D plus BPA.

The evaluation of gene expression in subcutaneous adipocytes showed significant inhibition of apoptosis by vitamin D. Findings from diet-induced obese mice confirmed that increased vitamin D intake due to induced apoptosis was negatively correlated to the volume of white adipose tissue. Apoptosis was prohibited in 3T3-L1 differentiated cells in a low dose of vitamin D, although apoptosis promoted by the high dose of vitamin D. Vitamin D in physiological dose suppresses mitochondrial uncoupling protein-2 (UCP2), enhanced mitochondrial potential and adenosine triphosphate (ATP) production, and repressed apoptosis [7]. It is postulated that a steady rise in intracellular calcium induced apoptosis by activating calpain-dependent calcium protease followed by activating caspase-12. In mature adipocytes, vitamin D stimulated voltage-dependent and non-voltage-dependent Ca^2+^ fluxes through Inositol 1,4,5-trisphosphate receptor/Ca^2+^ release channels (IP3R) and Ryanodine receptor/Ca^2+^ release channels (RyR) [7].

Contradictory, vitamin D can prevent lipogenesis in 3T3-L1 cells [25–27]. Kong et al. showed that vitamin D significantly reduced the lipid-filling rate in adipose tissue and moderated rats’ body weight. They suggested that vitamin D administration modified lipogenesis. They found that the protein levels of lipogenic factors, FAS, Stearoyl-CoA desaturase-1 (SCD1), and Acetyl-CoA carboxylase-1 (ACC1), were downregulated by vitamin D in adipose tissue of pregnant rats, the expression of SREBP-1c and C/EBP-α did not significantly decrease in adipose tissue. Moreover, PPAR-γ was markedly upregulated by vitamin D in adipose tissue [28].

The exposure to BPA is associated to the activation of C/EBPα, LPL, and mTOR, involved in lipogenesis and secretion of adipokines. BPA increases the expression of IL-6 and INF-γ which activates proinflammatory pathway JNK/STAT3/NFκB [17]. On the other hand, C/EBPβ induction happens early during the differentiation which involves mitotic clonal expansion and moderates the downstream upregulation of PPARγ and C/EBPα expression [28, 29]. In contrast to our results, Kong et al. presented that vitamin D had no effect on the expression level of C/EBPβ mRNA in the early stage of adipocyte differentiation. Consequently, they concluded that vitamin D suppression of PPARγ and C/EBPα depends on C/EBPβ expression. INSIG2 is recognized as an endoplasmic reticulum protein which binds to SREBP cleavage-activating protein and inhibits SREBPs activation. The INSIG2 mRNA levels were considerably upregulated following treatment with vitamin D in adipose tissue. Our results also showed 0.1 nM vitamin D plus BPA overexpressed INSIG2 mRNA through differentiation [28].

Consistent to our findings, several evidence [29, 30] indicated that vitamin D antagonizes the transacting activity of PPARγ by competing for the limited amount of RXR by VDR. When vitamin D binds and activates VDR, it may sequester RXR from PPARγ in the early stage of adipogenesis. VDR overexpression happens around C/EBPβ induction time. Thus, vitamin D will possibly inhibit adipocyte differentiation by preventing the reduction of VDR concentration in the late stages.

In contrast to our results which demonstrate an upregulation of adipogenic enzymes, FASN and LPL mRNA in the early stage accompanied by a decrease in the expression of FASN and LPL in the late stage of differentiation, Nimitphong et al. [23] showed that vitamin D increased LPL mRNA only during the later phase of adipogenesis in 3T3-L1.

The data indicated that BPA mediates as an obesogene by signaling through the activation of ER [23, 31–34].

Ohlstein et al. [20] pointed out that BPA increased induction of ER and adipogenic genes C/EBPα, insulin-like growth factor 1 (IFG1), PPARγ, and LPL during differentiation of hASCs into adipocytes. While, the additional studies indicated that BPA exposure promoted the expression of LPL [34, 35] and PPARγ [35, 36]. Chamorro-Garcia et al. [37] reported no effect in human BMSCs upon BPA treatment. Besides, Linehan et al. indicated that by decreasing LPL mRNA expression, BPA prevents lipid accumulation in human stem cells. The expression level of C/EBPα, PPARγ, and FABP4 were not significantly changed despite the upregulation of LPL through treatment with BPA, indicating that LPL can enhance the lipid accumulation independently of C/EBPα, PPARγ, and FABP4 y[21].

## Conclusions

One of the limitations of the present experiment is that lipid accumulation was not quantitatively measured during the morphological evaluation of differentiated cells. However, more lipid vacuoles were observed in 10 nM vitamin D plus BPA group compared to control and 0.1 nM vitamin D plus BPA groups.

To conclude, experimental studies identified the molecular targets and signaling pathways which may play a role in the pathogenesis of obesity due to exposure to BPA through examining tissues such as the thyroid, pancreas, liver, and adipose. BPA, as an endocrine disruptor, may be involved in lipid metabolism, enhance, and accelerate adipogenesis.

The effects of vitamin D and VDR on adipogenesis are not exactly the same, and VDR acts as an enhancer of adipogenesis, the role of vitamin D is not specified. It is currently challenging to determine the role of vitamin D in adipogenesis. Further researches are guaranteed to clarify its effect on adipogenesis.

## Data Availability

Not applicable.

## Abbreviations

ACC1: Acetyl-CoA carboxylase-1
ATP: Adenosine triphosphate
BMI: Body mass index
BPA: Bisphenol-A
C/EBP α: CCAAT-enhancer-binding protein-alpha
C/EBP β: CCAAT-enhancer-binding protein-Beta
DMEM: Dulbecco’s modified Eagle medium
ELISA: Enzyme-linked immunosorbent assay
ER-β: Estrogen receptor-beta
ETO: Eight-Twenty-One factor
FABP4: Fatty acid-binding protein-4
FASN: Fatty acid synthase
GAPDH: Glyceraldehyde-3-phosphate dehydrogenase
GLUT-1: Glucose transporter-1
GLUT4: Glucose transporter-4
hADMSCs: Human adipose-derived mesenchymal stem cells
IBMX: 3-Isobutyl-3-methylxanthine
IFG1: Insulin-like growth factor-1
INSIG2: Insulin- induced gene-2
IP3R: Inositol 1,4,5-trisphosphate receptor/Ca^2^^+^ release channels
LPL: Lipoprotein lipase
mRNA: Messenger ribonucleic acid
PBS: Phosphate-buffered saline
PPARγ: Peroxisome proliferator-activated receptor-gamma
qPCR: Quantitative polymerase chain reaction
RXR: Retinoid X receptor
RyR: Ryanodine receptor/Ca^2^^+^ release channels
SCD1: Stearoyl-CoA desaturase-1
SD: Standard deviation
SREBP1c: Sterol regulatory element-binding protein-1c
UCP2: Uncoupling protein-2
VDR: Vitamin D receptor
1,25(OH)2D3: 1,25-Dihydroxy-vitamin D3
